# Congenital coronary artery fistulae: a rare cause of heart failure in adults

**DOI:** 10.1186/1749-8090-9-87

**Published:** 2014-05-16

**Authors:** Charles M Geller, Kamellia R Dimitrova, Darryl M Hoffman, Robert F Tranbaugh

**Affiliations:** 1 Division of Cardiac Surgery, Mount Sinai Beth Israel, 317 East 17th Street, Fierman Building, 11 floor, New York, NY 10003, USA

**Keywords:** Coronary artery fistula, Heart failure

## Abstract

Coronary artery fistulae are uncommon, reported in 0.25% of patients undergoing coronary angiography. Two patients with congenital coronary artery fistula and coronary artery disease who presented with symptoms of exacerbated congestive heart failure out of proportion to their atherosclerotic burden were successfully treated by epicardial fistula ligation and coronary artery bypass grafting with marked improvement in functional status.

## Background

According to Ogden’s classification of congenital anomalies of the coronary arteries [1], coronary artery fistulae (CAF) are an anomaly of termination, representing abnormal communication between the coronary arteries and the cardiac chambers (coronary-cameral fistulae) or low-pressure veins (coronary arteriovenous malformations). Coronary artery fistulas were first described by Krause in 1865 [2] and are visualized in approximately 1 of 500 patients undergoing catheterization [3].

CAF may be congenital or acquired (infectious, traumatic or iatrogenic). Anomalies associated with congenital CAF include tetralogy of Fallot, atrial septal defect, patent ductus arteriosus, ventricular septal defect, pulmonary atresia with intact ventricular septum and superimposed coronary artery disease (35%) [4]. They also may be acquired secondary to trauma or iatrogenic interventions such as pacemaker implantation, endomyocardial biopsy, coronary artery bypass grafting, or coronary angiography (5). CAF may arise from any branch of the coronary artery system and originate from the right coronary artery (RCA) in 60%, left anterior descending (LAD) artery in 35%, and from the right posterior descending artery (RPDA), obtuse marginal (OM) and diagonal (D) arteries in 0.5% to 1.9% of patients (6). In congenital fistulae, drainage is most often to a low pressure cardiac chamber; the right ventricle (RV), right atrium (RA), or the pulmonary arteries (PA) and less frequently to the superior vena cava, coronary sinus, and pulmonary veins [3-6]. Communication into the left ventricle (LV) is extremely rare. The angiographic classification of Sakakibara [7] divides CAF into two categories-type A, proximal coronary segment dilated to the origin of the fistula with distal end normal and type B, coronary dilated over entire length, terminating as a fistula in the right side of the heart (end-artery type). Our first patient had an RPDA-to-LV coronary artery fistula and the second patient had a left main artery-to-PA fistula. Management of CAF in combination with obstructive coronary artery disease and dilated cardiomyopathy presenting with severe congestive heart failure is a unique surgical challenge.

## Case presentation

### Patient 1

A 68 year old African American male presented with exertional dyspnea, presyncope and chest pain. His past medical history was significant for hypertension, hypercholesterolemia, insulin dependent diabetes mellitus, and chronic atrial fibrillation, treated in the past with an unsuccessful atrioventricular nodal ablation and a permanent pacemaker. Chest x-ray showed severe cardiomegaly. Stress echocardiography revealed ischemia of the inferior myocardial wall. Transesophageal echocardiography demonstrated severe dilatation of the LV and reduced LV systolic function with an estimated ejection fraction (EF) of 12%. Coronary angiography revealed a RPDA fistula draining into the LV during diastole with dynamic compression of the fistula orifice during systole and significant lesions of both the LAD and the D. Utilizing normothermic cardiopulmonary bypass (CPB) with intermittent antegrade and retrograde cold blood cardioplegia, he underwent suture ligation of the fistulous vessel which was identified on the postero-inferior epicardial surface of the heart at the most distal segment of the RPDA near the interventricular septum. Additionally, coronary artery bypass grafting (CABG) was performed using the left internal thoracic artery (LITA) to bypass the LAD and a reversed saphenous vein graft (SVG) to bypass the D plus a left atrial cryoMaze procedure. After the ligation of the fistula, intraoperative transesophageal echocardiography showed a significant reduction in LV end diastolic volume.

The patient had an uncomplicated postoperative course and subsequently reported markedly increased exercise tolerance. One month after the surgery, transthoracic echocardiography exam showed improvement in EF to 35% and both LV diameter and volume. One year postoperatively, he was asymptomatic with excellent exercise capacity.

### Patient 2

A 59 year old Asian male presented with generalized fatigue and dyspnea on exertion. His past medical history was significant for congestive heart failure, hypertension and hypercholesterolemia. Chest x-ray showed severe cardiomegaly. Stress echocardiography was markedly positive. Transthoracic echocardiography demonstrated severe dilation of the LV and reduced LV systolic function with an estimated EF of 8%. Coronary angiography revealed a distal LM coronary artery fistula draining into the main PA and significant triple vessel coronary artery disease. Utilizing normothermic CPB with intermittent antegrade and retrograde cold blood cardioplegia, he underwent suture ligation of the fistulous vessel which was identified in the transverse sinus near the base of the left atrial appendage. Additionally, CABG was performed with LITA to LAD, SVG to PDA, and radial artery graft to an OM. He had an unremarkable post operative course. An echocardiogram prior to discharge revealed an EF of 25% and the patient was discharged with a wearable automatic defibrillator. He has subsequently reported markedly increased exercise tolerance.

## Conclusions

The etiology of congenital CAF remains obscure. The primitive coronary circulation consists of wide, endothelial-lined spaces between the muscle columns of the embryonal heart and of endothelial outgrowths towards the epicardial surfaces. The intertrabecular spaces or sinusoids freely communicate with these newly formed epicardial vessels and together form the original sinusoidal circulation [8]. Intracardiac coronary artery termination may be into the capillary plexus, into a myocardial sinusoid or directly into a ventricle. In a normal heart, the outermost intertrabecular spaces shrink and fuse with the coronary vessels to form the capillary network, and the intertrabecular vessels retain their ventricular communications to form the thebesian veins of the adult heart. When the intertrabecular spaces connecting coronary arteries, veins and cardiac chambers do not close, it give rise to persistent sinusoidal trabeculae, which may further develop into a CAF. With increased flow, the coronary artery branches proximal to the shunt site become significantly enlarged.

CAF tend to grow with age, and if untreated, fistulae cause clinical symptoms in 19% of affected patients <20 years of age and in 63% of older patients. Symptoms and sequelae are variable and include chronic myocardial ischemia and angina, congestive heart failure, cardiomyopathy, myocardial infarction, pulmonary hypertension, infective endocarditis, arrhythmias and rarely rupture. When detected in older patients CAF are often found in combination with atherosclerotic coronary artery disease [5], and/or after previous cardiac interventional procedures [9]. Frequently, as was the case with our patients, it is not clear which entity provoked the initial complaints and is likely a combination of both pathologies.

The physiology of the clinical picture depends on the resistance of the fistulous connection and on the site of fistula termination. The resistance is determined by the size, tortuosity, and length of the pathway. Flow from a coronary artery to a venous structure or right-sided cardiac chamber, occurs throughout the cardiac cycle. Blood follows the lower-resistance pathway through the fistulae rather than traversing the smaller arterioles and capillaries of the myocardium. With larger fistulae, a “diastolic runoff” may occur, drawing blood away from the normal coronary pathway with a widened pulse pressure and a coronary “steal”. A left-to-right shunt occurs if a fistula drains to the venous side of the circulation. When the drainage site is the LA, LV or PV, there is volume overloading of the left heart only.

Our two patients’ heart failure symptoms and dilated cardiomyopathy were attributed to the high output fistula causing significant LV volume overload, a hemodynamic situation resembling chronic aortic regurgitation.

Clinical symptoms of ischemia or volume overload, such as exertional angina or dyspnea, are the primary indication for closure of a fistula. The treatment of asymptomatic lesions is controversial, with some authors recommending early surgical intervention while others recommend a more conservative approach [10,11]. A symptomatic CAF can be treated by percutaneous transcatheter occlusion or suture obliteration [12]. The choice of treatment method depends on the anatomy and morphologic features of the fistula. In 1947 Bjork and Crafoord in 1947 [13] described the first successful surgical repair of a CAF. Twelve years later Swan et al. [14] reported the closure of a fistula using cardiopulmonary bypass. The first report of percutaneous therapeutic embolization was in 1983 when Reidy and colleagues [15] reported successful transcatheter fistula occlusion. Various transcatheter occlusion techniques have been used with excellent outcomes for fistulas with shorter, less tortuous courses. Our patients were not amenable to transcatheter closure because of their tortuous fistula anatomy and the presence of occlusive coronary artery disease.

Surgical closure can be done on a beating heart or on cardiopulmonary bypass (CPB) especially when it represents the termination of coronary artery branch. Proximal type CAF is treated by epicardial ligation, distal to the fistula origin, maintaining normal branch flow and can by done via either technique. Distal type CAF require ligation of the precapillary end by intracardiac purse-string suture at the site of termination and requires CPB.

Surgical treatment results in a low mortality from 0 to 4% and morbidity from 10% to 15% [16-18]. Operative correction in patients younger than 10 years or older than 45 years of age is coupled with an increase risk of morbidity and mortality. In a review done by McNamara et al. [19], 57% of patients were treated surgically with a 0.5% operative mortality rate. Cheung et al. [20] strongly advocated intra-cardiac closure of the fistula whenever possible because of the higher recurrence rate with the external plication technique.

Although the CAF in our patients were of the distal type, we chose to avoid a cardiotomy on a heart with already severely diminished left ventricular function.

While CAF are rare anomalies and those presenting in late adult life are even more unusual, CAF should always be considered in the differential diagnosis and diagnostic evaluation of patients with coronary artery disease and concomitant severely diminished systolic function due to dilated left ventricular cardiomyopathy. Surgical coronary revascularization and operative fistula ligation can markedly improve ventricular function and symptomatology.

## Consent

Written informed consent was obtained from the patients for the publication of this case report. A copy of the written consent is available for review by the Editor-in-Chief of this journal.

## Abbreviations

CAF: Coronary artery fistulae; RCA: Right coronary artery; LAD: Left anterior descending; RPDA: Right posterior descending artery; OM: Obtuse marginal artery; D: Diagonal artery; RV: Right ventricle; RA: Right atrium; PA: Pulmonary arteries; LV: Left ventricle; EF: Ejection fraction; CPB: Cardiopulmonary bypass; CABG: Coronary artery bypass graft; LITA: Left internal thoracic artery; SVG: Saphenous vein graft.

## Competing interests

The authors declare that they have no competing interests.

## Authors’ contributions

All authors 1) have made substantial contributions to conception and design, or acquisition of data, or analysis and interpretation of data; 2) have been involved in drafting the manuscript or revising it critically for important intellectual content; 3) have given final approval of the version to be published; and 4) agree to be accountable for all aspects of the work in ensuring that questions related to the accuracy or integrity of any part of the work are appropriately investigated and resolved. All authors read and approved the final manuscript.
